# Prevalence of overweight and obesity in United Arab Emirates Expatriates: the UAE National Diabetes and Lifestyle Study

**DOI:** 10.1186/s13098-017-0287-0

**Published:** 2017-11-02

**Authors:** Nabil Sulaiman, Salah Elbadawi, Amal Hussein, Salah Abusnana, Abdulrazzag Madani, Maisoon Mairghani, Fatheya Alawadi, Ahmad Sulaiman, Paul Zimmet, Oliver Huse, Jonathan Shaw, Anna Peeters

**Affiliations:** 1 Department of Family and Community Medicine, College of Medicine, University of Sharjah, P.O. Box 27272, Sharjah, UAE; 2 Dubai Health Authority, Dubai, UAE; 3 Rashid Centre for Diabetes and Research, Ajman, UAE; 4 Dubai Hospital, Dubai, UAE; 5 Royal College of Surgeons, Dublin, Ireland; 6 College of Medicine, University of New South Wales (UNSW), Sydney, Australia; 7 Baker/IDI, Melbourne, VIC Australia; 8 School of Health and Social Development, Deakin University, Geelong, VIC Australia

**Keywords:** United Arab Emirates, Expatriates, Obesity, Overweight, Prevalence, Adults

## Abstract

**Objective:**

To describe current prevalence of obesity and related non-communicable diseases (NCDs) in expatriates living in the United Arab Emirates (UAE).

**Methods:**

We used data from the cross-sectional UAE National Diabetes and Lifestyle Study (UAEDIAB), which surveyed adult expatriates living in the UAE for at least 4 years. We report crude prevalence of overweight and obesity, indicated by gender and ethnicity-specific body mass index (BMI), waist circumference (WC) and waist-to-hip ratio (WHR) cut-offs, by lifestyle and biomedical characteristics, as well as age and sex-adjusted odds ratios.

**Results:**

Out of a total of 3064 recruited expatriates (response rate 68%), 2724 had completed all stages of the UAEDIAB study. Expatriates were; 81% men, mean age 38 years (range 18–80), 71% South East Asians, and 36% university graduates. In this sample, the prevalence of overweight and obesity, by BMI, were 43.0 and 32.3%, respectively. 52.4 and 56.5% of participants were at a substantially increased risk according to WC and WHR, respectively. The prevalence of diabetes, hypertension and hypercholesterolemia were 15.5, 31.8, and 51.7%, respectively, with the prevalence of each being higher in those with obesity.

**Conclusion:**

Prevalence of obesity and associated NCDs are extremely high in UAE expatriates. Without comprehensive prevention and management, levels of disease will continue to increase and productivity will fall.

## Background

In 1997, The World Health Organization (WHO) Expert consultation on Obesity warned that populations of most countries would be facing an obesity epidemic that will put them at risk of developing non-communicable diseases (NCDs). Overweight and obesity prevalence has continued to increase rapidly since that date creating a true pandemic and now affecting younger age groups [1]. Worldwide, the proportion of adults with a body-mass index (BMI) of 25 kg/m^2^ or greater increased between 1980 and 2013 from 28.8 to 36.9% in males, and from 29.8 to 38.0% in females [2]. Further, the NCD risk factor collaboration [3] predicts that these increasing trends will continue into the future.

In nations of the Gulf Cooperation Council (GCC) (Bahrain, Kuwait, Qatar, Oman, Saudi Arabia and the UAE), in 2011, an increase in per-capita income as a result of vast population and economic growth, coupled with increasing life expectancy and decreasing mortality from communicable diseases has resulted in increasing rates of obesity and associated NCDs [4]. The prevalence of overweight in GCC adults has been reported to be 48% amongst males and 35% amongst females, while the prevalence of obesity has been reported to be 24% amongst males and 40% amongst females [5]. Further, there have been increases in the prevalence of overweight and obesity in GCC states in recent times. More specific to the UAE, one survey, conducted over 15 years ago (1999–2000) found that a third of the population was obese and over 40% overweight [6]. In this survey, UAE citizens were purposely oversampled compared to the expatriate population. As the population of the UAE is predominantly made up of expatriates [7], it is likely that by under-sampling this population group, the true prevalence of overweight and obesity in the UAE has not been fully captured.

Almost all recent studies in the GCC focus on Emirati nationals and fail to consider the prevalence in the expatriates who considerably outnumber the nationals [7]. Therefore, the objective of this study is to determine the recent prevalence of overweight and obesity and the factors associated with it, in the UAE expatriate population using different anthropometric measurements and population-specific cut-offs.

## Methods

This study used data from the first phase of the UAE National Diabetes and Lifestyle (UAEDIAB) study, which is described in detail elsewhere [8]. In brief, this was a two-phase cross-sectional survey of all non-pregnant and non-institutionalised adult (aged ≥ 18 years) expatriates who had resided in the UAE for at least 4 years, designed to investigate the prevalence of diabetes and its associated risk factors in Emiratis and expatriates living in the UAE. The first phase of the study (used here) sampled expatriates who have been living for at least 4 years in the UAE and the second phase sampled the whole UAE population and has been ongoing since its commencement in May 2014.

The first phase of the UAEDIAB study was conducted between the years 2012 and 2014 using an innovative, cost effective, random and representative sample of residents in Dubai, Sharjah and the Northern Emirates [8]. Abu Dhabi is the only emirate not participating in the UAEDIAB study, as a comprehensive population survey is already in place in this emirate. In the UAE, expatriates visit Preventive Medicine Departments (PMDs) every 2–3 years for periodic medical examinations as a legal requirement for the renewal of their residence visa [8]. Therefore, PMDs provided an ideal opportunity to obtain a representative sample. The UAE National Bureau of Statistics estimated the sample size and the selected PMDs in each Emirate. In each PMD, a systematic random sampling was adopted whereby every 10th eligible visitor was invited to participate. A 68% response led to a total of 3064 participating expatriates [8]. Those who consented participated in a 30 min structured interview on general demographics, including; ethnicity, education and age, as well as lifestyle characteristics and attitudes. Following this, physical measurements, including height, weight, waist, hip and neck circumferences and blood pressure, were taken. The specific methods used in taking these measurements are described in detail in a later section. Finally, blood samples were drawn and tested for fasting blood glucose, HbA1c, lipids and cholesterol at a reference laboratory (Rashid Centre for Diabetes and Research, Ajman, UAE). Blood samples were centrifuged within 4 h of being drawn, before being transported in an insulated icebox to the laboratory for testing [8]. Of the 3064 expatriates who were recruited for the UAEDIAB Study, 2724 completed all stages of data collection. Table 1 shows the demographic characteristics of the study population.

**Table 1:** Study population characteristics for the total population, and for males and females separately

Variable	Total population (2724)	Males (2204)	Females (520)
BMI^a^			
Normal	21.1% (n = 575)	20.2% (n = 445)	25.2% (n = 130)
Overweight	43.0% (n = 1172)	44.7% (n = 986)	35.5% (n = 183)
Obese	32.3% (n = 881)	31.4% (n = 691)	36.5% (n = 188)
WC^a^			
Substantially increased risk of metabolic complications	52.4% (n = 1428)	50.8% (n = 1120)	59.8% (n = 308)
WHR^a^			
Substantially increased risk of metabolic complications	56.5% (n = 1538)	59.2% (n = 1305)	45.2% (n = 233)
Age			
Mean age in years	38 (SD = 10.3)	37.8 (SD = 10.0)	38.6 (SD = 11.8)
Ethnicity			
Asian	63.4% (n = 1727)	67.0% (1476)	48.0% (n = 247)
Arabic	23.2% (n = 633)	20.6% (n = 455)	34.6% (n = 178)
Western/African	3.1% (n = 84)	2.3% (n = 51)	6.4% (n = 33)
Marital status			
Single	18.5% (n = 504)	18.5% (n = 407)	18.6% (96)
Married	80.2% (n = 2184)	80.9% (n = 1782)	77.5 (n = 399)
Divorced/widowed/separated	1.2% (34)	0.6% (n = 14)	3.9 (n = 20)
Education			
Never attended school	14.2% (n = 386)	15.0% (n = 330)	10.7% (n = 55)
Some high school	37.4% (n = 1019)	40.0% (n = 881)	26.6% (n = 137)
Diploma	12.6% (n = 342)	12.1% (n = 266)	14.8% (n = 76)
University bachelor	27.8% (n = 756)	25.5% (n = 561)	37.1% (n = 194)
Post grad	8.1% (n = 220)	7.5% (n = 166)	10.3% (n = 53)
Income (AED per year)			
< 36,000	39.8% (n = 1085)	41.0% (n = 904)	34.6% (n = 178)
36,000–95,999	26.9% (n = 732)	26.9% (n = 593)	27.0% (n = 139)
96,000–179,999	16.0% (n = 436)	16.3% (n = 359)	14.8% (n = 76)
≥ 180,000	11.7% (n = 319)	11.3% (n = 250)	13.4% (n = 69)
Watch TV for less than 1 h daily			
No	58.4% (n = 1591)	58.4% (n = 1288)	58.1% (n = 299)
Yes	39.6% (n = 1078)	39.3% (n = 866)	41.0% (n = 211)
Current smoker			
No	79.7% (n = 2172)	76.9% (n = 1695)	91.8% (n = 473)
Yes	20.2% (n = 551)	23.1% (n = 509)	8.2% (n = 42)
Physical activity			
No	53.0% (n = 1440)	53.9% (n = 1187)	49.1% (n = 253)
Yes	47.0% (n = 1279)	46.1% (n = 1017)	50.9% (n = 262)

Results are presented as means, with standard deviations, for continuous variables and as proportions for categorical variables

*AED* United Arab Emirates Dirham

^a^Defined through ethnicity-specific cutoffs

All neck, hip and waist measurements were taken using a non-stretchable plastic tape. Waist circumference was measured at the midpoint between the lower margin of the lowest palpable rib and the top of the iliac crest. Hip circumference was measured around the widest portion of the buttocks, with the tape parallel to the floor. The neck circumference was measured at the midpoint of the neck’s height, with participants standing upright. Weight and height were measured using a certified SECA stadiometer and weighing scale. For all measurements, the participant had to stand still with feet close together, arms at the side and would wear little clothing and no shoes. Each measurement was repeated three times and in the case that the measurements are within 1 cm of one another, the average was calculated. In case the difference between the three measurements exceeded 1 cm, the three measurements were repeated.

Body mass index (BMI) was calculated as weight (kg)/height (m^2^). Cut-points used to define overweight and obesity differed by ethnicity. Among Arabs and Europids, a BMI of 25 to 30 kg/m^2^ indicated overweight and ≥ 30 kg/m^2^ indicated obesity [9]. Asians with a BMI of 23 to < 27.5 kg/m^2^ were considered overweight while a BMI value of ≥ 27.5 kg/m^2^ indicated obesity [10]. Waist circumference (WC) cut-points indicating substantially increased risk of metabolic complications differed by ethnicity and gender. Amongst Arabs this was ≥ 102 cm for males and ≥ 88 cm for females, amongst Asians ≥ 90 cm for males and ≥ 80 cm for females, and amongst Europids; ≥ 94 cm for males and ≥ 80 cm for females [11]. In all ethnic groups, waist-to-hip ratio (WHR) more than or equal to 0.90 and 0.85 indicated substantially increased risk for males and females respectively [11].

Age was classified as mean age, in years. Participants’ ethnicity was defined as either Asian, Arabic or Western/African, while marital status was defined as single, divorced/widowed/married or separated. Participants’ education was classified as; ‘never attended school’, ‘some high school education’, ‘diploma qualification’, ‘a university bachelor degree’ or ‘a post graduate degree’. Participants’ income, in UAE Dirham per year, was divided into < 36,000, 36,000–95,999, 96,000–179,999 or ≥ 180,000. Participants’ responses were classified as either ‘yes’ or ‘no’ to each of; ‘are you a current smoker’, ‘do you watch TV for more than 1 h a day’ and ‘do you regularly perform physical activity (for transport or recreation/leisure purposes)’.

Data is also available for medical conditions. The presence of diabetes was indicated by Fasting Plasma Glucose (FPG) ≥ 7.0 mmol/L, and Hemoglobin A1C (HbA1C) ≥ 6.5% [12]. Hypertension was given by systolic blood pressure ≥ 140 mmHg and/or diastolic blood pressure ≥ 90 mmHg [13]. High total cholesterol was indicated by ≥ 5.0 mmol/L, high high-density lipoprotein (HDL) cholesterol was represented by ≥ 1 mmol/L for men and ≥ 1.3 mmol/L for women, while low-density lipoprotein (LDL) cholesterol was categorised as ‘<2.59 mmol/L’, ‘2.59–3.34 mmol/L’ and ‘>3.34 mmol/L’ [14]. High triglyceride levels were indicated by ≥ 1.7 mmol/L [14]. Finally, participants responded as either ‘yes’ or ‘no’ to suffering snoring or sleep apnea.

### Statistical analysis

For baseline population characteristics, univariate analyses reported means and standard deviations (SD) for continuous variables, and proportions for categorical variables. Overall prevalence of obesity was also reported after direct standardization to adjust crude prevalence rates by age and sex using the world mid-year population of 2013 [15].

Logistic regression was used to estimate the associations between indicators of obesity and dichotomous demographic and health variables (gender, TV viewing habits, smoking status, presence of diabetes, presence of hypertension, high total, HDL or LDL cholesterol levels, high triglyceride levels, presence of snoring, and presence of sleep apnea), adjusting for age and sex.

The level of significance was set at 5% (*p* < 0.05) and accordingly confidence intervals were calculated with a 95% level of confidence. Statistical analysis was performed using SPSS (Version 23).

## Results

To avoid loss of data, participants with missing data for any variables of interest were not excluded from analyses (n = 2724). Table 1 shows the demographic characteristics of the study population.

The crude prevalence of overweight and obesity as measured by BMI was 43.0 and 32.3%, respectively. When standardized to the age and sex structure of the 2013 world mid-year population, the prevalence for overweight and obesity were 37.5% (95% CI of 35.7–39.3%) and 34.9% (95%CI of 33.1–36.7%) respectively. According to WC and WHR measurements, 52.4 and 56.5% of the study population, respectively, were classified as substantially increased risk. When standardized to the age and sex structure of the 2013 world mid-year population, obesity prevalence as measured by WC was 53.8% (95% CI of 51.9–55.6%) and for that measured by WHR was 50.0% (95% CI of 48.1–51.9%).

Table 2 shows the crude prevalence of obesity by BMI, WC and WHR, alongside results from logistic regression analyses, adjusted for age and sex. The prevalence and odds of obesity by BMI, WC and WHR increased with increasing age. According to BMI and WC, females were significantly more likely to be obese than males [OR = 1.24 (95% CI 1.01–1.53) and 1.69 (95% CI 1.35–2.12), respectively], while by WHR, females were significantly less likely to be obese than males (OR = 0.52, 95% CI 0.42–0.65). According to BMI, Arabic expatriates were significantly more likely to be obese (prevalence of obesity = 40.7%) compared to Westerners/Africans (22.6%) and Asians (30.5%). By WC, Asian expatriates were significantly more likely to be obese than Arabic expatriates (OR = 2.24, 95% CI 1.84–2.73). By WHR, Western/African expatriates were significantly less likely to be obese than Arabic expatriates [OR = 0.48 (95% CI 0.30–0.79)]. Married participants were more likely to be obese than those who were single [OR = 1.57 (95% CI 1.19–2.05)], but those who were divorced/widowed/separated were not significantly more likely to be obese than those who were single [OR = 1.47 (95% CI 0.68–3.14)]. Current smokers and those who watched TV for less than 1 h a day were more likely than never smokers and those who watched TV for more than 1 h a day to have an increased BMI [OR = 1.32 (95% CI 1.08–1.62) and 1.37 (95% CI 1.16–1.63), respectively], but neither smoking status or TV viewing time were significantly related to WC or WHR. There was no significant association of either income or education with WC or WHR. Those who performed some physical activity were more likely to be obese by BMI than those who did not [OR = 1.27 (95% CI 1.08–1.502)], though the opposite association was observed for WHR [OR = 0.73 (95% CI 0.61–0.87)] and no significant association was seen for WC [OR = 0.97 (95% CI 0.82–1.15)].

**Table 2:** Prevalence and risk of obesity, as measured by BMI, WC, and WHR, by socio-demographic factors

Variable	BMI^a^		WC^a^		WHR	
	Obesity prevalence (%)	Risk of obesity (odds ratio, 95% CI)^c^	Obesity prevalence (%)	Risk of obesity (odds ratio, 95% CI)^c^	Obesity prevalence (%)	Risk of obesity (odds ratio, 95% CI)^c^
Age						
18–30	23.2	Reference group	40.4	Reference group	44.7	Reference group
31–40	30.6	1.48 (1.18–1.85)	59.5	2.19 (1.78–2.70)	64.9	2.31 (1.87–2.84)
41–50	41.0	2.35 (1.84–2.99)	69.0	3.39 (2.65–4.32)	75.0	3.68 (2.86–4.73)
51–60	51.0	3.43 (2.53–4.65)	74.9	4.44 (3.15–6.25)	74.3	3.68 (2.62–5.18)
61 +	46.2	2.78 (1.72–4.51)	78.9	5.25 (2.89–9.51)	83.1	6.86 (3.59–13.11)
Gender						
Male	32.6	Reference group	56.8	Reference group	66.2	Reference group
Female	37.5	1.24 (1.01–1.53)	68.1	1.69 (1.35–2.12)	51.7	0.52 (0.42–0.65)
Ethnicity						
Arabs non-nationals	40.7	Reference group	47.5	Reference group	62.5	Reference group
Asians non-Arabs	30.5	0.66 (0.54–0.80)	63.0	2.24 (1.84–2.73)	64.8	1.05 (0.86–1.28)
Westerners and Africans	22.6	0.41 (0.24–0.71)	59.8	1.57 (0.96–2.56)	45.1	0.48 (0.30–0.79)
Marital status						
Single	20.9	Reference group	33.9	Reference group	44.0	Reference group
Married	36.4	1.57 (1.19–2.05)	64.6	2.40 (1.87–3.08)	67.9	1.61 (1.26–2.06)
Divorced/widowed/separated	39.4	1.47 (0.68–3.14)	60.0	1.40 (0.64–3.11)	70.0	1.86 (0.81–4.31)
Education						
Never attended School	33.1	Reference group	62.1	Reference group	71.7	Reference group
Some high school	30.7	0.99 (0.77–1.29)	56.7	0.91 (0.70–1.18)	66.7	0.89 (0.67–1.17)
Diploma	32.4	1.07 (0.77–1.48)	56.4	0.84 (0.61–1.17)	61.3	0.73 (0.52–1.03)
University bachelor	37.7	1.37 (1.05–1.80)	61.1	1.05 (0.79–1.39)	57.5	0.65 (0.48–0.86)
Post grad	35.0	1.10 (0.77–1.58)	59.4	0.84 (0.58–1.21)	56.9	0.54 (0.37–0.79)
Income (AED^b^ per year)						
< 36,000	28.2	Reference group	57.1	Reference group	64.5	Reference group
36,000–95,999	35.5	1.39 (1.12–1.71)	58.4	0.97 (0.79–1.20)	61.1	0.82 (0.66–1.01)
96,000–179,999	40.8	1.64 (1.29–2.09)	59.4	0.96 (0.75–1.24)	63.0	0.83 (0.64–1.07)
≥ 180,000	35.7	1.21 (0.92–1.60)	65.4	1.12 (0.84–1.49)	66.4	0.88 (0.66–1.19)
Watch TV for less than 1 h daily						
No	30.8	Reference group	58.0	Reference group	64.2	Reference group
Yes	38.4	1.37 (1.16–1.63)	60.3	1.07 (0.89–1.28)	61.3	0.86 (0.72–1.02)
Smoking						
No	20.7	Reference group	59.8	Reference group	63.1	Reference group
Yes	28.7	1.32 (1.08–1.62)	55.2	0.90 (0.73–1.12)	65.3	1.04 (0.84–1.30)
Physical activity						
No	30.9	Reference group	59.2	Reference group	66.9	Reference group
Yes	36.5	1.27 (1.08–1.50)	58.5	0.97 (0.82–1.15)	59.5	0.73 (0.61–0.87)

*AED* United Arab Emirates Dirham

^a^Defined through ethnicity-specific cutoffs

^b^For WC and WHR, non-substantially increased risk and substantially increased risk categories are referred to as non-obese and obese, respectively

^c^Adjusted for age and sex

Table 3 shows the association between excess bodyweight, as measured by BMI, WC and WHR, and a number of relevant health conditions. All health conditions (except for sleep apnea with WC), were higher in participants who were classified as substantially increased risk by BMI, WC or WHR. Of those with obesity by BMI, 23.5% had diabetes by FPG (≥ 7 mmol/L) [OR = 1.81 (95% CI 1.44–2.27)], 17.4% had diabetes by HbA1C (≥ 6.5%) [OR = 2.13 (95% CI 1.63–2.78)], 38.5% had hypertension [OR = 1.39 (95% CI 1.16–1.66)], 56.5% had high total cholesterol [OR = 1.27 (95% CI 1.07–1.50)] and 45.6% had high triglyceride levels [OR = 1.72 (95% CI 1.45–2.05)]. Similar results were seen amongst those with substantially increased risk by WC and WHR.

**Table 3:** Prevalence and risk of health condition by obesity, as measured by BMI, WC and WHR

Variable/obesity group	BMI^a^		WC^a^		WHR	
	Prevalence (%, n)	Risk of health condition (odds ratio–95% CI)^b^	Prevalence (%, n)	Risk of health condition (odds ratio–95% CI)^b^	Prevalence (%, n)	Risk of health condition (odds ratio–95% CI)^b^
Diabetes (FPG ≥ 7 mmol/L)						
Non-obese	11.8 (205)	Reference group	10.7 (106)	Reference group	9.8 (86)	Reference group
Obese	23.5 (207)	1.81 (1.44–2.27)	19.5 (277)	1.51 (1.17–1.95)	19.3 (296)	1.68 (1.28–2.21)
Diabetes (HbA1C ≥ 6.5)						
Non-obese	6.9 (121)	Reference group	6.1 (61)	Reference group	4.9 (43)	Reference group
Obese	17.4 (153)	2.13 (1.63–2.78)	13.6 (193)	1.67 (1.22–2.29)	13.7 (211)	2.20 (1.54–3.14)
Hypertension (Sys ≥ 140 and/or Dias ≥ 90)						
Non-obese	28.4 (494)	Reference group	23.9 (237)	Reference group	22.2 (195)	Reference group
Obese	38.5 (338)	1.39 (1.16–1.66)	38.0 (541)	1.72 (1.42–2.09)	37.9 (582)	1.63 (1.33–1.98)
Total cholesterol (≥ 5.0)						
Non-obese	49.2 (859)	Reference group	45.0 (457)	Reference group	45.9 (405)	Reference group
Obese	56.5 (498)	1.27 (1.07–1.50)	55.0 (785)	1.29 (1.09–1.5)	54.3 (835)	1.245 (1.05–1.48)
Low HDL (< 1 for males and < 1.3 for females)						
Non-obese	33.9 (592)	Reference group	31.2 (311)	Reference group	30.0 (265)	Reference group
Obese	43.2 (380)	1.449 (1.26–1.77)	40.1 (572)	1.47 (1.23–1.76)	40.2 (618)	1.63 (1.36–1.96)
LDL (> 3.34)						
Non-obese	43.6 (761)	Reference group	41.4 (413)	Reference group	39.4 (348)	Reference group
Obese	51.4 (453)	1.33 (1.13–1.58)	49.3 (704)	1.30 (1.10–1.55)	49.9 (767)	1.34 (1.12–1.59)
Triglycerides (≥ 1.7)						
Non-obese	32.3 (565)	Reference group	28.0 (279)	Reference group	26.7 (236)	Reference group
Obese	45.6 (401)	1.72 (1.45–2.05)	42.2 (602)	1.85 (1.54–2.22)	41.9 (644)	1.67 (1.38–2.02)
Snoring						
Non-obese	14.1 (227)	Reference group	13.0 (120)	Reference group	14.7 (123)	Reference group
Obese	32.0 (265)	2.52 (2.05–3.11)	23.9 (315)	1.74 (1.37–2.21)	22.1 (311)	1.28 (1.01–1.63)
Sleep apnea						
Non-obese	1.8 (31)	Reference group	2.5 (24)	Reference group	1.5 (13)	Reference group
Obese	4.4 (38)	2.28 (1.39–3.73)	25 (35)	0.90 (0.52–1.56)	3.1 (46)	2.02 (1.06–3.85)

Percentages are calculated within obesity categories

For WC and WHR, non-substantially increased risk and substantially increased risk categories are referred to as non-obese and obese, respectively

^a^Defined through ethnicity-specific cutoffs

^b^Adjusted for age and sex

## Discussion

This study revealed a high prevalence of overweight and obesity (43.0 and 32.3%, respectively), by BMI, amongst expatriate residents in the UAE in 2013. The prevalence of a substantially increased risk of metabolic complications, by WC and WHR, was also high (52.4 and 56.5%, respectively). Females were more likely to be obese than males and the highest obesity rates were seen in Arabic non-Emiratis. Those with an increased BMI, WC and WHR were significantly more likely to face adverse health outcomes, including diabetes, hypertension and high cholesterol.

Previous studies have also examined the cross-sectional prevalence of overweight and obesity in the UAE [6, 16, 17]. Most comparably, Malik et al. [6] reported the prevalence of overweight and obesity (41 and 33%, respectively) in the UAE multiethnic population, in the year 2000. The prevalence of obesity was lowest amongst UAE nationals compared to other ethnicities, though exact data were not reported for UAE nationals and expatriates separately. Females showed a higher prevalence of obesity than males (40 and 24%, respectively) but a lower prevalence of overweight (35 and 48%, respectively), according to BMI. Such a pattern was also observed in the present study, and previous studies conducted in the UAE have also demonstrated women to have a higher prevalence of obesity and a lower prevalence of overweight compared to men [4–6]. This gender-patterning of the prevalence of overweight and obesity has been observed in previous studies conducted in the Middle East [18]. Our results showed that Arabs had the highest prevalence of obesity (40.7%), compared to other ethnicities, when BMI was used as the indicator of adiposity, though this was not the case when WC and WHR were used. Supporting this, Malik et al. [6] reported that, whilst obesity was common within all ethnic groups, there was a higher prevalence of obesity amongst Egyptian and North African expatriates (48%), compared to other ethnicities. Differences in obesity by BMI, WC and WHR between ethnicities have also been reported elsewhere [19], lending further credibility to the results observed here. Evidence is mixed regarding ethnicity specific cut-offs for non-Europeans [19]. This provides one possible explanation for the observed differences in obesity by BMI, WC and WHR between ethnicities in the present study. Malik et al. [6] conducted their previous study over 15 years ago, and UAE citizens were purposely oversampled compared to the expatriate population, which is not representative of the actual UAE population [7]. Therefore, while it is possible to compare point-prevalence data from the previous study and our study, it is difficult to estimate trends from this available data.

A recent study [16] which examined the prevalence of overweight and obesity amongst both Emirati (n = 1035) and expatriate (n = 405) schoolchildren in the UAE showed that 14.7% of students were overweight and 18.9% were obese and 61.3% of students had a BMI percentile that was above the 50th CDC percentile [16]. In another study of UAE school children [17], the prevalence of overweight was found to range from 11.5 to 41.2% in age groups from 3 to 6 years until 15–18 years, while the prevalence of obesity was found to range between 5.2 and 19.3% in age groups from 3 to 6 years until 15–18 years. While the focus on schoolchildren prevents comparisons between these two previous studies and the present study, they provide further indication of the severity of overweight and obesity levels in the UAE.

There are several possible drivers of these patterns of high prevalence of overweight, obesity and metabolic conditions in expatriates in the UAE. There is not a strong culture of physical activity in the Gulf States [20], potentially due to the climate, and this could be a causative factor for high overweight and obesity prevalence. In particular, women are known to have especially low physical activity levels due to social norms and a lack of facilities [21], and this could explain the comparatively high prevalence of obesity amongst women. Other factors that could contribute to a high prevalence of overweight, obesity and metabolic conditions include a high prevalence of smoking, emotional stress and maid use [4]. However, a lack of longitudinal studies in the UAE prevents causality from being determined.

As would be expected, the present study reported higher rates of metabolic disorders amongst overweight and obese participants, compared to normal participants. Previous research into such disorders in the UAE has recognised that diabetes is a serious health concern here, with a self-reported type 2 diabetes prevalence of 11.3% amongst a population comprising UAE nationals and expatriates [4]. The UAE has also been noted to have the highest prevalence of hypertension amongst GCC states, with the self-reported prevalence reaching 13.3% in the year 2000. The prevalence of these conditions was increased amongst overweight and obese individuals [4]. Further, the prevalence of both type 2 diabetes and hypertension was reported to have increased at a greater rate than all other GCC states between 1995 and 2000 [4]. This was supported by other research across the GCC states, indicating that the prevalence of both diabetes and high cholesterol levels is increasing across the region [5]. However, the limited number of available studies prevents concrete conclusions on the trends in these metabolic disorders from being made.

Previous research has strongly indicated that in high income countries, socioeconomic position (SEP) and obesity are inversely related, such that as SEP decreases, the prevalence of overweight and obesity increases [22, 23]. Conversely, in the present study, the prevalence of overweight and obesity increased with increasing income and highest achieved education. However, evidence also exists showing that, in less developed and developing nations, those of a higher socioeconomic position tend to exhibit a greater prevalence of overweight and obesity [22–24]. Amongst lower income households, an association has also been observed between increasing household income and increasing BMI [25]. This provides support for the results seen in the present study, as the majority of UAE expatriates included in the study population were Asian or Arabic in descent, where a significant number of developing countries are located [26].

One of the surprising results from the present study was that the prevalence of overweight and obesity by BMI was higher amongst those who performed physical activity and watched TV for less than 1 h a day, though such relationships were not observed for other indicators of adiposity. Previous research has strongly indicated that increased physical activity and decreased TV viewing has a protective effect against excess adiposity [27]. The observed relationship between physical activity/TV viewing time and overweight and obesity could be due to overweight and obese participants actively trying to lose weight. This indicates the importance of raising awareness when it comes to healthy dietary patterns and supporting physical activity in the UAE population.

The main strength of this study was the rich data on a wide range of biomedical and sociodemographic characteristics for a moderately large sample size (n = 3064). However, there are also some limitations to this analysis. First, the first phase of the UAEDIAB Study (used here) targeted expatriates to the UAE only, and so UAE nationals could not be included in the analysis. However, as the population of the UAE is predominantly made up of expatriates [7], this limitation is of only modest impact. Further, previous studies suggest that UAE expatriates present a higher prevalence of obesity than UAE citizens [6], and so the present study could be surveying an at-risk group. Second, compared to the UAE expatriate population, more men responded to the UAEDIAB Study than women [8], resulting in some sampling bias. However, this would likely be corrected by age and gender standardization. Third, the expatriates included in this study, have likely spent most of their lives living elsewhere and could well have been overweight or obese prior to arriving in the UAE. Further, as a cross-sectional survey, the present study is unable to accurately determine the causative factors surrounding overweight and obesity in the UAE. Future studies would do well to examine the trends in overweight, obesity and the associated health outcomes, in the UAE.

Overweight and obesity is a major health problem in expatriates living in UAE. It is associated with diabetes, hypertension, high lipids and cholesterol as well as snoring and sleep apnea. In order to accurately monitor trends, there is a need for longitudinal studies. To monitor the onset and progress of the diabetes, the UAE has recently developed a new diabetes registry. Similar surveillance programs should be implemented for other metabolic conditions as well. Policies should be targeted in attempts to prevent obesity but it is important to understand the various environmental factors affecting health outcomes and without proper cohort and longitudinal studies this may prove difficult. The prevalence of overweight and obesity in the UAE is alarming and longitudinal monitoring strategies and preventative interventions should be implemented.
